# Consensus recommendations of three-dimensional visualization for diagnosis and management of liver diseases

**DOI:** 10.1007/s12072-020-10052-y

**Published:** 2020-07-07

**Authors:** Chihua Fang, Jihyun An, Antonio Bruno, Xiujun Cai, Jia Fan, Jiro Fujimoto, Rita Golfieri, Xishan Hao, Hongchi Jiang, Long R. Jiao, Anand V. Kulkarni, Hauke Lang, Cosmas Rinaldi A. Lesmana, Qiang Li, Lianxin Liu, Yingbin Liu, Wanyee Lau, Qiping Lu, Kwan Man, Hitoshi Maruyama, Cristina Mosconi, Necati Örmeci, Michael Pavlides, Guilherme Rezende, Joo Hyun Sohn, Sombat Treeprasertsuk, Valérie Vilgrain, Hao Wen, Sai Wen, Xianyao Quan, Rafael Ximenes, Yinmo Yang, Bixiang Zhang, Weiqi Zhang, Peng Zhang, Shaoxiang Zhang, Xiaolong Qi

**Affiliations:** 1 The First Department of Hepatobiliary Surgery, Zhujiang Hospital, Southern Medical University, Guangdong Provincial Clinical and Engineering Center of Digital Medicine, Guangzhou, 510282 China; 2 Department of Gastroenterology, Hanyang University College of Medicine and Hanyang University Guri Hospital, Guri, 11923 South Korea; 3 Department of Experimental, Diagnostic and Specialty Medicine-DIMES, University of Bologna, S. Orsola-Malpighi Hospital, Via Giuseppe Massarenti 9, 40138 Bologna, Italy; 4 Department of General Surgery, Sir Run Run Shaw Hospital, School of Medicine, Zhejiang University, Hangzhou, Zhejiang China; 5 Department of Liver Surgery and Transplantation, Liver Cancer Institute, Zhongshan Hospital, Key Laboratory of Carcinogenesis and Cancer Invasion of Ministry of Education, Fudan University, Shanghai, 200032 China; 6 Key Laboratory of Medical Epigenetics and Metabolism, Institutes of Biomedical Sciences, Fudan University, Shanghai, 200032 China; 7 Department of Surgery, Hyogo College of Medicine, 1-1 Mukogawa-cho, Nishinomiya, Hyogo 663-8501 Japan; 8 Department of Gastrointestinal Cancer Biology, Tianjin Medical University Cancer Institute and Hospital, National Clinical Research Center for Cancer, Key Laboratory of Cancer Prevention and Therapy, Tianjin’s Clinical Research Center for Cancer, Tianjin, China; 9 Department of Liver Surgery, The First Affiliated Hospital Harbin Medical University, Harbin, 150001 Heilongjiang China; 10 HPB Surgical Unit, Department of Surgery and Cancer, Imperial College, London, W12 0HS UK; 11 Department of Hepatology, Asian Institute of Gastroenterology, Hyderabad, India; 12 Department of General, Visceral and Transplantation Surgery, University Medical Center of the Johannes Gutenberg-University, Langenbeckst. 1, 55131 Mainz, Germany; 13 Division of Hepatobiliary, Department of Internal Medicine, Faculty of Medicine, Universitas Indonesia, Cipto Mangunkusumo National General Hospital, Jakarta, 10430 Indonesia; 14 National Clinical Research Center for Cancer and Key Laboratory of Cancer Prevention and Therapy, Tianjin Medical University Cancer Institute and Hospital, Tianjin, 300060 China; 15 Department of Hepatobillirary Surgery, the First Affiliated Hospital of USTC, Division of Life Sciences and Medicine, University of Science and Technology of China, Hefei, 230001 Anhui China; 16 Department of General Surgery, Xinhua Hospital Affiliated To Shanghai Jiao Tong University School of Medicine, Shanghai, China; 17 Faculty of Medicine, The Chinese University of Hong Kong, Hong Kong, China; 18 Department of General Surgery, Central theater General Hospital of the Chinese people’s Liberation Army, Wuhan, 430070 Hubei China; 19 Department of Surgery, LKS Faculty of Medicine, University of Hong Kong, Hong Kong, China; 20 Department of Gastroenterology, Juntendo University, 2-1-1, Hongo, Bunkyo-ku, Tokyo, 113-8421 Japan; 21 Department of Gastroenterology, Ankara University Medical School, Ibn’i Sina Hospital, Sihhiye, 06100 Ankara, Turkey; 22 Oxford NIHR Biomedical Research Centre, University of Oxford, Oxford, UK; 23 Internal Medicine Department, Federal University of Rio de Janeiro (UFRJ), Rio de Janeiro, RJ Brazil; 24 Division of Gastroenterology, Department of Medicine, Faculty of Medicine, Chulalongkorn University and King Chulalongkorn Memorial Hospital, Bangkok, 10700 Thailand; 25 Department of Radiology, Assistance-Publique Hôpitaux de Paris, APHP, HUPNVS, Hôpital Beaujon, 100 bd du Général Leclerc, 92110 Clichy, France; 26 Department of Hydatid & Hepatobiliary Surgery, Digestive and Vascular Surgery Centre, First Affiliated Hospital of Xinjiang Medical University, Urumqi, 830054 China; 27 Department of Radiology, Zhujiang Hospital, Southern Medical University, Guangzhou, 510282 China; 28 Department of Gastroenterology, University of Sao Paulo School of Medicine, Sao Paulo, Brazil; 29 Department of General Surgery, Peking University First Hospital, Beijing, China; 30 Department of Surgery, Hepatic Surgery Center, Tongji Hospital, Tongji Medical College, Huazhong University of Science and Technology, Wuhan, China; 31 Institute of Digital Medicine, School of Biomedical Engineering and Medical Imaging, Army Medical University (Third Military Medical University), Chongqing, 400038 China; 32 CHESS Center, Institute of Portal Hypertension, The First Hospital of Lanzhou University, Lanzhou, China

**Keywords:** Three-dimensional visualization, Computed tomography, Quality control system, Three-dimensional printing, Hepatocellular carcinoma, Hilar cholangiocarcinoma, Hepatolithiasis, Portal hypertension, Living donor liver transplantation, Consensus

## Abstract

Three-dimensional (3D) visualization involves feature extraction and 3D reconstruction of CT images using a computer processing technology. It is a tool for displaying, describing, and interpreting 3D anatomy and morphological features of organs, thus providing intuitive, stereoscopic, and accurate methods for clinical decision-making. It has played an increasingly significant role in the diagnosis and management of liver diseases. Over the last decade, it has been proven safe and effective to use 3D simulation software for pre-hepatectomy assessment, virtual hepatectomy, and measurement of liver volumes in blood flow areas of the portal vein; meanwhile, the use of 3D models in combination with hydrodynamic analysis has become a novel non-invasive method for diagnosis and detection of portal hypertension. We herein describe the progress of research on 3D visualization, its workflow, current situation, challenges, opportunities, and its capacity to improve clinical decision-making, emphasizing its utility for patients with liver diseases. Current advances in modern imaging technologies have promised a further increase in diagnostic efficacy of liver diseases. For example, complex internal anatomy of the liver and detailed morphological features of liver lesions can be reflected from CT-based 3D models. A meta-analysis reported that the application of 3D visualization technology in the diagnosis and management of primary hepatocellular carcinoma has significant or extremely significant differences over the control group in terms of intraoperative blood loss, postoperative complications, recovery of postoperative liver function, operation time, hospitalization time, and tumor recurrence on short-term follow-up. However, the acquisition of high-quality CT images and the use of these images for 3D visualization processing lack a unified standard, quality control system, and homogeneity, which might hinder the evaluation of application efficacy in different clinical centers, causing enormous inconvenience to clinical practice and scientific research. Therefore, rigorous operating guidelines and quality control systems need to be established for 3D visualization of liver to develop it to become a mature technology. Herein, we provide recommendations for the research on diagnosis and management of 3D visualization in liver diseases to meet this urgent need in this research field.

## Introduction

The application and advance of digital intelligent diagnostic and treatment technology in liver surgery [1] have facilitated 3D visualization of liver to be the most effective method helping surgeons thoroughly to comprehend the complex internal anatomy of the liver [2, 3]; it brings about revolutionary changes to the accurate diagnosis and management of liver diseases [4–6]. By means of this technology, individualized display of both complex internal anatomy of liver and spatial information of liver and its surrounding organs enables surgeons to “see more”, to “see better”, and to “see more accurately”; it also enables surgeons to obtain relatively comprehensive information available for assisting clinical decision-making [7]. In the past, clinicians used to transform 2D information into abstract 3D models in their minds with the aid of their personal experience. Nonetheless, the limitation and uncertainty of their experience may lead to an uncertain and inconsistent reconstruction outcome. While 3D reconstructed models or 3D printed models can visualize and intuitively display variations of intrahepatic blood vessels, and provide a convenient and accurate method for liver volume calculation, virtual simulation surgery, and surgical navigation [8, 9]. Moreover, the non-invasive evaluation of hepatic venous pressure gradient (HVPG) using quantitative imaging, combined with 3D model and hemodynamics, is a promising method for the diagnosis and monitoring of portal hypertension [10, 11].

The development of 3D simulation software for liver surgery permitted surgeons to perform surgical planning and virtual hepatectomy on 3D models [12]. Subsequent advances in technology improved the 3D simulation software to estimate liver volume based on portal venous blood flow, and studies have shown a clear correlation between the simulated hepatectomy and actual hepatectomy volumes [8, 13]. Since 2012, 3D image-assisted liver surgery has been covered by the national health insurance in Japan, affirming the role of 3D preoperative simulation for liver surgery [14]. In China, the Digital Medicine Branch of the Chinese Medical Association has reached expert consensuses and guidelines on the application of 3D visualization technology (3DVT) for the accurate diagnosis and treatment of complicated liver tumors, hilar cholangiocarcinoma, hepatolithiasis, pancreatic head cancer, gallbladder carcinoma, and retroperitoneal tumors, as well as application of computer-assisted indocyanine green fluorescence imaging technology in the diagnosis and surgical navigation of liver tumors [15], forming standardized protocols for the use of 3D visualization [16]. At present, 3D simulation software systems have received widespread acceptance in terms of preoperative evaluation, surgical planning, and intraoperative navigation of liver diseases [5, 17, 18]. There are many alternative 3D reconstruction software programs available in the market that can be used for liver surgery simulation, such as HepaVision (Mevis, Germany) [19, 20], Liver Analyzer (Mevis, Germany) [21], MI-3DVS (Southern Medical University, China) [7], 3D VSP and VR-Anat (IRCAD, France) [22], Scout Liver and Explorer Liver (Pathfinder Technologies, USA) [23], SYNAPSE VINCENT (Fujifilm Medical, Japan) [24], Virtual Place (AZE, Japan) [25], and Hitachi Image Processing System (Hitachi Medical, Japan) [26], etc.

Three-dimensional visualization has a great potential in displaying lesion information and guiding diagnosis and treatment. At meta-analysis, the application of 3D visualization technology in the diagnosis and management of primary hepatocellular carcinoma was found to have extremely significant differences in reducing (a) intraoperative blood loss, (b) postoperative complications, (c) operating time and hospitalization time, and (d) recurrence rate of liver cancer in short-term follow-up and accelerating the recovery of postoperative liver function significantly [27]. There is, however, inevitable heterogeneity due to the differences in diseases and operations by different surgeons [28, 29]. Thus, it remains a main challenge to acquire high-quality CT images, and conduct 3D reconstruction and visualization analysis using homogeneous, standardized, and quality controlled systems to achieve comprehensive reflection of lesion information by 3D models that could assist physicians in clinical decision-making. The previous reports on 3D visualization mainly focus on how 3D models have changed clinical practice of liver diseases [5, 29]; however, the reconstruction of these models lacks a homogeneous and standardized processing, and clinical consistency and practical evaluation. Therefore, these models possibly with reconstruction errors may not be suitable for clinical practice.

In this consensus, we describe the workflow of 3D visualization along with its latest advances in the diagnosis and management of liver diseases. The opportunities and challenges presented by 3D visualization to improve individualized accurate diagnosis and management of liver diseases are highlighted, with an emphasis on the importance of homogeneous and standardized 3D reconstruction process and quality control system. Finally, we envision the future development direction and new opportunities of 3D visualization in clinical practice.

This consensus refers to the Grading of Recommendations Assessment, Development and Evaluation (GRADE), which divides the quality of evidence into high, moderate, and low or very low levels. These levels of evidence are reported with the letter grades of A, B, and C, respectively (Table 1) [30–32]. The strength of recommendation, formed by GRADE grid method (Table 1), was divided into strong (1) and weak (2) recommendations. Participated experts voted to determine the strength of recommendation based on the quality of evidence, patient values, and preferences.

**Table 1:** Quality of evidence and strength of recommendations

Grade	Classification	Content
Quality of evidence		
High	A	We are very confidence that the true effect lies close to that of the estimated effect
Moderate	B	We are moderately confident in the effect estimate: the true effect is likely to be close to the estimated effect, but there is a possibility that it is substantially different
Low or very low	C	Our confidence in the effect estimate is limited: the true effect may be substantially different from the estimated effect; we have very little confidence in the effect estimate: the true effect is likely to be substantially different from the estimated effect
Strength of recommendation		
Strong recommendation	1	The desirable effects outweigh the undesirable effects
Weak recommendation	2	The desirable effects possibly outweigh the undesirable effects

## The workflow of 3D visualization

**Fig. 1:**
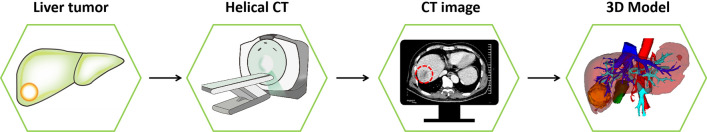
Flowchart depicting the workflow of 3D visualization in hepatology. CT-based 3D images can display the spatial relationship between the lesion and the intrahepatic vascular system stereoscopically and intuitively, which can improve the surgeon’s comprehension of the disease, and provide critical information for clinical decision-making

**Fig. 2:**
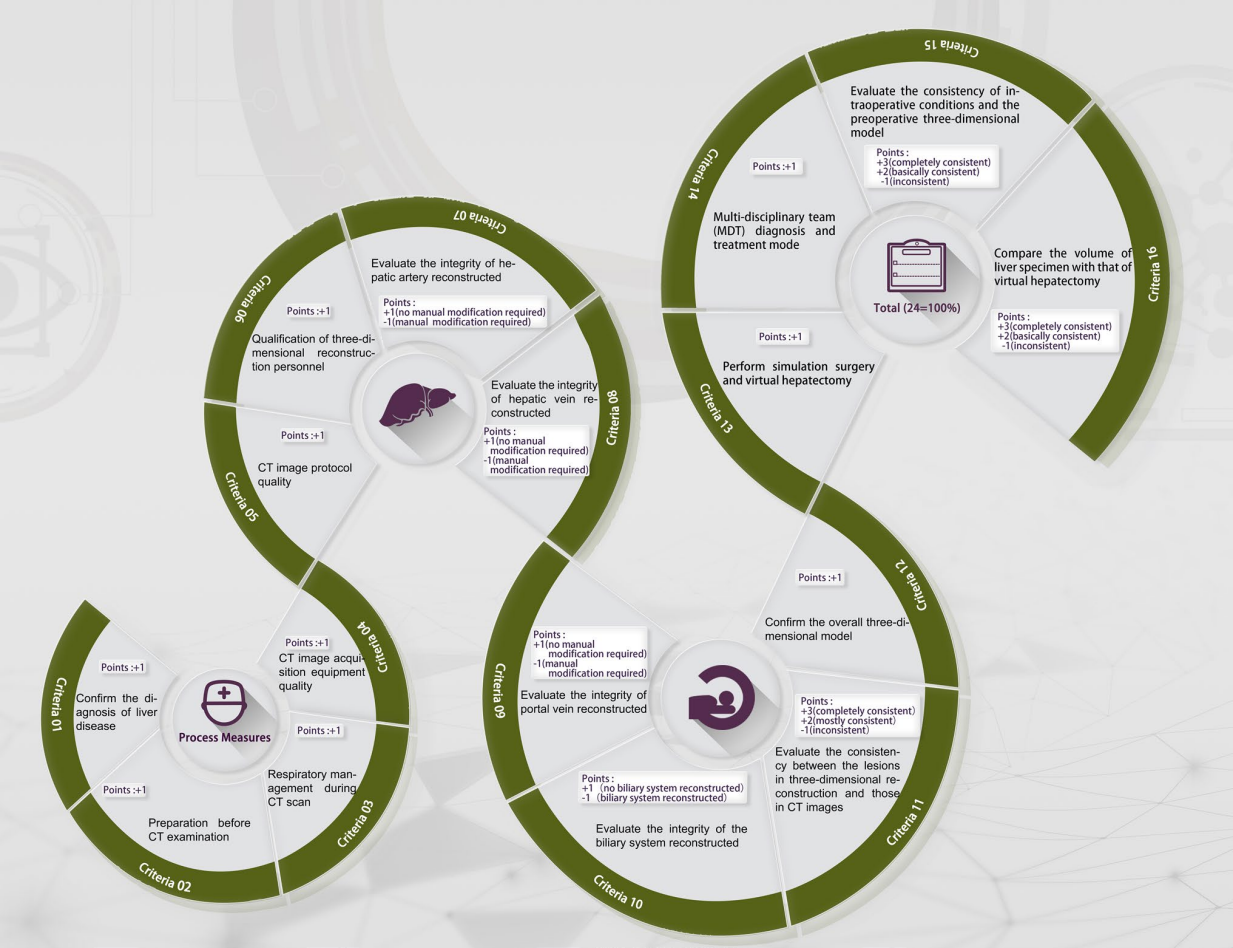
Flowchart depicting the workflow of the application of the 3DVQS. The workflow includes necessary steps in a 3D visualization analysis. The 3DVQS both rewards and penalizes the methodology and analyses of a study, consequently encouraging optimal and standardized clinical practice

3DVT refers to the visualization of human body information reflected on CT images into 3D effects on a computer, thus providing structural information unable to be obtained from 2D images (Fig. 1). 3D visualization processing mainly includes the following steps: CT data acquisition, image processing and 3D reconstruction, image registration, and fusion and visualization analysis. For complicated liver diseases, noticeably, 3D reconstruction can be performed based on fusion imaging combining MRI venous phase and CT venous phase [2]. To evaluate and guarantee the quality of 3DVT, we have established the quality control system and 3D visualization quality score (3DVQS) (Fig. 2).

## Acquisition of CT data

A routine plain CT should be performed in the supine position with a scan range from the top of the diaphragm to the lower level of both kidneys to ensure coverage of the entire liver and the portal vein. The scanning range should be adjusted according to each patient’s clinical status, and the scanning parameters should be set according to the specific CT scanner. Dynamic phases in the setting of different liver pathologies should be noted. For patients with hepatocellular carcinoma, attention should be paid to the image quality of arterial phase, portal venous phase, and delayed phase, and for those with intrahepatic cholangiocarcinoma and hilar cholangiocarcinoma in particular, attention should be paid to the image quality of delayed phase; for those with hepatolithiasis, attention should be paid to image quality of plain scan phase and portal venous phase. Multiphase images (plain scan phase, arterial phase, portal venous phase, and delayed phase) should be obtained by a 64-slice or above helical CT scanner, with a section thickness of 0.625–1 mm. Data should be archived in the DICOM format and exported by storage equipment. CT parameters: cross-sectional scanning, tube voltage 100–140 kV, or set the optimal voltage based on patient size and weight, slice thickness 0.625–1 mm, pitch 0.891–0.915, large scan field of view, standard reconstruction algorithm, and thin-layer reconstruction if necessary. Scanning procedure and protocol: routine plain scan of upper abdomen. Typically, a water-soluble iodine medium is administered by rapid bolus injection. For bolus injection, the usual dose is 1.0 to 2.0 ml/kg (1.5 ml/kg for obese patients) at an injection rate of 4.7 to 5.0 ml/s with scanning begun immediately after administration. Usually, triple-phase scanning should be adopted, i.e., arterial phase, portal venous phase, and delayed phase. The original scanning time is set based on plain scan, and the timing of the delay is synchronized with the start of injection. The arterial phase scan is triggered by the threshold triggering protocol or starts at a delay of 20–30 s. The portal venous and delayed scans start at a delay of 60–70 s and 2 min, respectively, from the beginning of contrast agent administration [17, 33, 34].

## Image processing and 3D visualization

The acquired DICOM data should be imported into a 3D reconstruction software for data segmentation. The original 2D images of abdominal organs, lesions, and vascular system can be reconstructed automatically, with the intrahepatic vascular branches reaching the level of 3–4. Based on the automatically generated liver contour and vascular model, at least one abdominal imaging attending and one attending physician should collaborate to determine, manually check and modify the lesion scope; in case of disagreement, negotiate and discuss or refer to another senior physician to ensure a more accurate 3D visualization image; this process is particularly important in the 3D reconstruction of some complicated diseases.

## Three-dimensional visualization

Based on the 3D models reconstructed by original CT images, the shape and spatial distribution of the target, such as the liver, biliary tract, blood vessels, tumors, or stones, can be defined and delineated; in addition, the target can be separated visually, accurately, and rapidly. Thus, the clinical decision for accurate preoperative diagnosis, individualized surgical planning, and selection of surgical approach can be provided [35]. 3D visualization mainly centers upon the following aspects:

Individualized evaluation of hepatic lesions: based on the 3D images, the size, morphology, and location of lesions (tumors or stones) can be evaluated, and the same is true for the evaluation of the anatomical relationship between lesions and intrahepatic vessels to guide surgical treatment [36].

Individualized evaluation of the morphology of biliary system with biliary diseases: individualized anatomical variation can be analyzed and evaluated based on the 3D visualization classification system for biliary system, which can display the biliary system stereoscopically. In the presence of hepatolithiasis, the size of stones and their distribution in each hepatic segment, and the extent and range of biliary stenosis can be clearly displayed [17, 37]. It is recommended to use minimum intensity projection (min MIP) processing with CT to improve biliary conspicuity. Patients with normal biliary system can be assessed and classified with MRI.

Individualized evaluation of the morphology of hepatic arterial system: according to Michel’s classification for hepatic artery variants [38], the origin of left and right hepatic arteries, the presence of accessory hepatic artery and other vascular variations should be evaluated based on 3D images; the hepatic artery variants should be evaluated according to the 3D classification system for hepatic artery.

Individualized evaluation of the morphology of hepatic venous system: the course and variation of hepatic vein should be evaluated based on the 3D classification system for hepatic vein. The variations of right posterior inferior hepatic vein, segment IV hepatic vein, and segment VIII hepatic vein have significant value in the decision-making of liver surgery [39, 40].

Individualized evaluation of the morphology of portal venous system: the anatomical variations of left branch, right branch and trunk of portal vein, splenic vein, and superior mesenteric vein should be evaluated according to the 3D classification system for portal vein [41].

Individualized liver segmentation and volume calculation: the individualized liver segmentation and volume calculation should be performed according to the topological relation of portal vein blood flow, and meanwhile, the volume calculation of any portal vein branch drainage area can be conducted. The optimal virtual resection plane should be determined according to the tumor location, together with the distance and spatial relationship between the tumor and the intrahepatic vascular system; the volume of the remaining functional liver should be calculated through simulation surgery, and meanwhile, postoperative integrity of venous drainage and portal vein blood supply of each hepatic segment retained should be ensured. For major liver resection or living liver donations, volume calculation is equally necessary [8, 42]

## Quality control system of 3DVT

To better apply 3DVT in the field of liver diseases, it is urgently needed to establish a standardized evaluation and homogeneous operation flow. We have put forward the quality control system and 3DVQS based on the following three critical criteria: preoperative 3D surgical simulation, intraoperative 3D surgical navigation, and postoperative 3D reconstruction; they have been further identified as 16 criteria, each of which is elaborated in Table 2 [15].

**Table 2:** Process Measures

Criteria		Points
1	Diagnosis of liver diseases by preoperative imaging (ultrasound, CT or MRI)	+ 1
2	Patients fast for at least 4 h prior to CT scan, orally take 0.5L–1.0L of clear liquid 20 to 30 min prior to the exam and take another 500 ml prior to the exam	+ 1
3	Train the patient to hold their breath in full inspiration before scanning and instruct them to do so during each scan phase to achieve maximum management of artifacts due to respiratory motion	+ 1
4	Select 64-slice or above spiral CT scanning with slice thickness of 0.625–1.0 mm	+ 1
5	CT scanning ranges from the top of the diaphragm to the lower level of both kidneys, and, furthermore, perform dynamic abdominal scan after intravenous contrast medium administration; perform CT celiac arteriography. The arterial phase, portal venous phase, and delayed phase scans start at a delay of 20–25 s, 50–55 s, and 2 min, respectively	+ 1
6	3D reconstruction should be performed by attending physicians or a level above who are engaged in the diagnosis and treatment of liver diseases	+ 1
7	Evaluate the integrity of the course, shape, and continuity of hepatic artery reconstructed by 3D visualization to determine whether manual revision is required (manual revision is unnecessary when the tertiary branches of artery can be reconstructed)	+ 1 (no manual revision); − 1 (manual revision required)
8	Evaluate the integrity of the course, morphology, and continuity of hepatic vein reconstructed by 3D visualization to determine whether manual revision is required (Manual revision is unnecessary if the tertiary branches of hepatic vein can be reconstructed)	+ 1 (no manual revision); − 1 (manual revision) required)
9	Evaluate the integrity of the course, morphology, and continuity of portal vein reconstructed by 3D visualization to determine whether manual revision is required. The branches of the portal vein system with the diameter ≥ 5 mm should be reconstructed (it is unnecessary if the tertiary branches of portal vein can be reconstructed)	+ 1 (no manual revision); − 1 (manual revision required)
10	Evaluate its course, morphology, continuity, and integrity of the 3D reconstructed biliary tract (manual revision is unnecessary if the tertiary branches of biliary tree can be reconstructed)	+ 1 (biliary system reconstructed); − 1 (no biliary system reconstructed)
11	Evaluate the morphology, size, and distribution of lesions in the 3D reconstructed model and whether they are consistent with CT images	+ 3(basically consistent, no manual revision required); + 2(mostly consistent, manual revision required); -1(inconsistent, manual revision required)
12	The overall 3D model should be validated by at least 2 abdominal imaging attendings and at least 2 attending hepatologists in comparison with the original CT images, and finally confirmed by a senior physician	+ 1
13	Perform simulation surgery based on 3D model. The simulation of various schemes should be carried out and the optimal surgical approach and surgical resection plane should be selected by two attending physicians, and finally confirmed by a senior physician	+ 2
14	A multi-disciplinary team (MDT) should be formed based on the individualized 3D model and the results of clinical examinations; liver surgeons undertake the main tasks, assisted by the departments of hepatology, oncology, endoscopy, interventional therapy, and radiotherapy	+ 2
15	The consistency between preoperative 3D models and intraoperative conditions (lesions, vascular variance, and range of hepatectomy) should be assessed	+ 3 (completely consistent); + 2 (basically consistent); − 1 (inconsistent)
16	The volume of the virtual resected liver with that of the actual resected liver (reference standard is intraoperative dewatering method) should be compared. The volume error (< 5%) is completely consistent, the volume error (< 10%) is basically consistent, and the volume error (> 10%) is inconsistent	+ 3 (completely consistent); + 2 (basically consistent); − 1 (inconsistent)

Total score (24 = 100%). The score no more than 15 is recognized as undesirable; the score more than 15 is recognized as desirable

Recommendation 1 Operator: Sufficient anatomical basis and solid knowledge of liver surgery are required, along with at least 30 cases of standardized operation training (B, Strong recommendation)

Recommendation 2 Examinee: The patient should fast for at least 4 h prior to CT exam, rest for 10–20 min, and hold their breath during scanning (B, Strong recommendation).

Recommendation 3 Homogeneous 3D model: It is suggested to select 64-slice or above helical scanner with a slice thickness of 0.625–1.0 mm for 3D reconstruction (A, Strong recommendation).

Recommendation 4 It is suggested that the original 3D model be repeatedly discussed, verified and modified by at least 2 abdominal imaging attendings and at least 2 attending physicians (A, Strong recommendation).

Recommendation 5 It is suggested that analysis of hepatic veins, hepatic arteries, portal veins, and bile ducts should be performed by at least 1 abdominal imaging attending and 1 attending physician for vascular system classification (A, Strong recommendation).

Recommendation 6 It is suggested to perform virtual simulation surgery using 3D model before operation, to select the optimal surgical approach and surgical resection plane, and calculate the residual functional liver volume on an individual basis (A, Strong recommendation).

Recommendation 7 It is suggested to determine the final surgical planning by combining the results of MDT discussion based on 3D visualization, as well as the wishes of patients and their family (A, Strong recommendation).

Recommendation 8 It is recommended to measure the volume of liver specimen and compare it with that of virtual resection to obtain the discrepancy between surgical planning and actual operation (A, Strong recommendation).

Recommendation 9 For patients with liver diseases diagnosed and treated by 3D visualization, it is recommended to follow the quality control steps of 3DVT and conduct quality scoring, which is conducive to standardize the effect of clinical evaluation (A, Strong recommendation).

## Criteria for quality scoring

At present, many studies on preoperative 3D simulated liver surgery have been reported; nevertheless, it is difficult to reach any homogenization due to the inconsistent quality of 3D models reconstructed by different 3D reconstruction software. Despite their certain automatic and intelligent characteristics, the liver contour and vascular model generated automatically still need to be manually checked and modified to minimize deviation and to improve the effectiveness of the 3D model [15].

Recommendation 10 It is necessary to follow the quality control steps and conduct quality scoring, no matter what 3D visualization software is used (A, Strong recommendation).

## Potential for application and promotion

The establishment of a homogeneous quality control system and 3D visualization quality score is helpful to standardize the application and promotion of 3DVT in the diagnosis and treatment of liver diseases. The core contents of homogenization research proposed by this consensus include acquisition of high-quality CT images, 3D reconstruction, simulated vascular resection and reconstruction, individualized liver volume calculation, and virtual hepatectomy. Therefore, it is suggested that a 3D reconstruction software should be used in institutions where condition permits to evaluate virtual hepatectomy to improve the safety of surgery [15].

## Diagnosis and management of complicated liver tumor by 3D visualization

Currently, the definition of complicated liver tumor is controversial [15, 43]; it generally refers to centrally located hepatocellular carcinoma involving the porta hepatis; variations of hepatic artery, portal vein, and hepatic vein within the liver; serious distortion of intrahepatic vessels due to massive compression of tumor; malignant liver tumors with inferior vena cava or even right atrial cancer thrombus; massive benign or malignant tumors of the liver requiring extensive hepatectomy; hepatic tumors involving hepatic segments I and VII that require complex hepatectomy. Individualized 3D reconstruction enables surgeons to visualize, describe, and analyze complex intrahepatic structures and anatomic relationships of tumors, especially in patients with large tumors or with tumor compression of important blood vessels requiring extended hepatectomy (≥ 3 segments) [44, 45], which is associated with a higher risk of postoperative liver failure. Thus, it is significant to conduct the accurate and comprehensive preoperative evaluation and reasonable formulation of surgical planning.

Recommendation 11 For patients with complicated liver tumors requiring hepatectomy, it is recommended that 3D visualization of portal vein classification be carried out to comprehend the course, variation, and its relationship with tumor (A, Strong recommendation).

## Classification of 3D visualization models for centrally located hepatocellular carcinoma

The central region of the liver mainly consists of hepatic segments IV, V, or VIII, the upper boundary of which is the junction of the three hepatic veins with the inferior vena cava, the lower boundary is the middle part of the anterior hepatic margin, the left margin is the falciform ligament, the right margin is the right intersectoral fissure, and the abaxial side, connected to the hepatic caudate lobe, is adjacent to the inferior vena cava and the first hepatic portal. Therefore, centrally located hepatocellular carcinoma can be defined as a liver cancer adjacent to the main hepatic branches, such as hepatic vein, portal vein, bile duct system, or retrohepatic inferior vena cava, or a liver cancer with a distance of less than 1 cm [46, 47].

3D visualization classification of centrally located hepatocellular carcinoma (Fang’s classification) can be divided into the five types [7] (Table 3).

**Table 3:** 3D Classification and surgical methods of centrally located hepatocellular carcinoma

Classification	Description	Surgical methods
	Type I: The tumor is in the liver parenchyma of segments V, VIII, or both**,** characterized by their close proximity to or even direct violation of the adjacent portal vein. They do not adhere to or compress the right hepatic vein trunk	Resection of segments V, VIII ± partial resection of segment IV
	Type II: The tumor is in the liver parenchyma of segments IV a, IV b, or both, characterized by its proximity to or even direct violation of the left hepatic vein trunk**.** In addition, it does not adhere to or compress the left hepatic vein trunk	Resection of segments IV a and IV b or left hepatectomy
	Type III: The tumor occupies most liver parenchyma of segments IV, V, and VIII, characterized by a wide and deep invasion of the parenchyma, or their proximity to the middle hepatic vein	Central bisectionectomy (resection of segments IV, V, and VIII ± I)
	Type IV: This type of liver tumor occupies most liver parenchyma of segments IV, V, and VIII, characterized by their close proximity to, or direct violation of, the left/right portal vein trunk or the left/right hepatic vein	Resection of segment IV, V, VI, VII, VIII Resection of segment II, III, IV, V, VIII Reduced right trisectionectomy or reduced left trisectionectomy Associating liver partition and portal vein ligation for staged hepatectomy (ALPPS)
	Type V: This type of liver tumor occupies the superficial liver parenchyma of segments IV, V and VIII. The lesions are not close to either the portal branch or the hepatic vein	Hepatectomy with a negative margin

Recommendation 12: For patients with a centrally located hepatocellular carcinoma, 3D visualization classification should be used to select the corresponding recommended surgical method (A, Strong recommendation).

## Three-dimensional visualization classification of complicated hepatocellular carcinoma with blood vessels as the axis

Hepatectomy mainly involves the portal vein, hepatic vein, inferior vena cava, and hepatic artery systems. Therefore, this consensus proposes a 3D visualization classification system for complicated hepatocellular carcinoma with blood vessels as the axis to guide surgery (Table 4) [47].

**Table 4:** Three-dimensional visualization classification of complicated hepatocellular carcinoma with blood vessels as the axis

Type	Grading
Type I: tumor involving portal vein	Grade 0: vessels are not compressed by tumors Grade 1: vessels are compressed but not invaded by tumors Grade 2: vessels are invaded but not interrupted by tumors Grade 3: tumor invasion with continuity interruption of blood vessels
Type I a: tumor involving the right branch of portal vein	
Type I b: tumor involving the left branch of portal vein	
Type II: tumor involving hepatic vein	
Type II a: tumor involving right hepatic vein	
Type II b: tumor involving middle hepatic vein	
Type II c: tumor involving left hepatic vein	
Type III: tumor involving hepatic artery	
Type III a: tumor involving right hepatic artery	
Type III b: tumor involving left hepatic artery	
Type IV: tumor involving inferior vena cava	
Type V: tumor involving abdominal aorta	
Type VI: other cases	

Recommendation 13: Perform digitalized classification, resectability evaluation, and surgical planning of blood vessels as the axis for complicated liver tumor using 3D visualization technology to achieve anatomical, functional, and radical surgical resection of complicated liver tumor (A, Strong recommendation).

## Guidance of 3DVT for other therapeutic methods of hepatocellular carcinoma

Transarterial chemoembolization (TACE) and transcatheter portal vein embolization (PVE) are considered useful treatment modalities for hepatocellular carcinoma [48]. By means of 3DVT, the main blood artery supply and its small branches supplying the tumor can be clearly displayed, and an accurate 3D "vessel-tumor" model can be provided for hepatic artery variations [49, 50]; 3D visualization of the main portal vein and its branches can be used to guide the compensated hepatic proliferation induced by portal vein embolization to increase the volume of functional liver [51]. 3DVT can also provide a preoperative 3D approach for percutaneous liver tumor radiofrequency ablation and argon–helium knife technology, and accurately measure the volume of intraoperative electrode probe failure range.

Recommendation 14: For patients undergoing TACE, tumor ablation, and argon–helium knife, 3D visualization technology should be used to evaluate the condition of hepatic artery (A, Strong recommendation).

## The application of 3D printing in complicated liver surgery

**Fig. 3:**
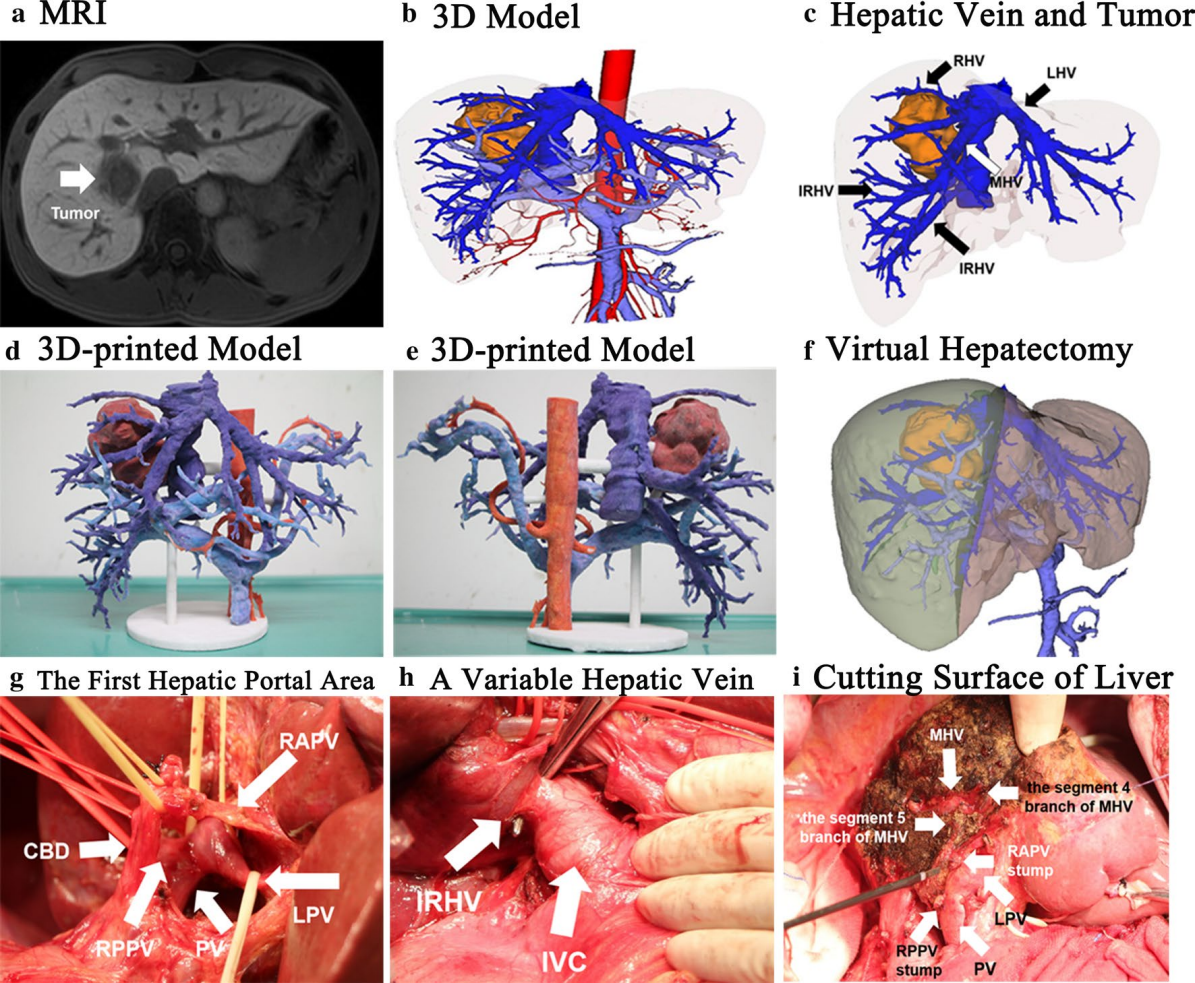
Complicated hepatectomy guided by 3D printing technology. A Type III variation of portal vein, inferior right hepatic vein (IRHV) flowing into IVC. **a** MRI showed that the tumor was located in the right-anterior liver; **b** CT-based 3D model; **c** 3D model showed the relationship between tumor and variant hepatic vein; **d** 3D printed model (anterior view); **e** 3D printed model (posterior view); **f** virtual hepatectomy; **g** portal hepatis structures; **h** variant IRHV flowing into IVC; **i** after tumor resection, the course of branches of middle hepatic vein on segments 4 or 5 and the transected ends of RAPV and RPPV can be seen. *LHV* left hepatic vein, *MHV* middle hepatic vein, *RHV* right hepatic vein, *IRHV* inferior right hepatic vein, *PV* portal vein, *LPV* left-portal vein, *RAPV* right-anterior portal vein, *RPPV* right posterior portal vein, *CBD* common bile duct, *IVC* inferior vena cava

3D reconstructed images can well show the 3D relationship between important blood vessels, biliary structure, and liver parenchyma. However, there is still a lack of authenticity in displaying 3D images on a 2D computer screen. Due to the lack of reliable liver surface markers, it is still difficult to achieve intraoperative guidance of 3D images; meanwhile, the morphological changes caused by traction of the liver and respiratory movement have been claimed to be the key constraints. These obstacles are expected to be overcome using high-precision 3D printing models [52, 53]. Figure 3 demonstrates the resection of a complex right hepatic tumor guided by 3D reconstruction in combination with 3D printing. Apart from reconstruction by a 3D visualization software, a further 3D printed model can truly restore the characteristics of the organs in vivo and approximate reality based on 3DVT. The advantages are described as follows [54–56]: (1) the location, size, and shape of the tumor can be faithfully represented, and the relationship between the tumor and vascular systems can be observed from different perspectives. (2) Real-time navigation can be provided during the operation, and vital positions can be identified and located quickly.

Recommendation 15: For patients with complicated liver tumors, preoperative 3D printed model might be used to guide the actual surgical procedure if the hospital has an appropriate post-processing workstation (B, Strong recommendation).

## Diagnosis and treatment of hilar cholangiocarcinoma guided by 3D visualization technology

**Fig. 4:**
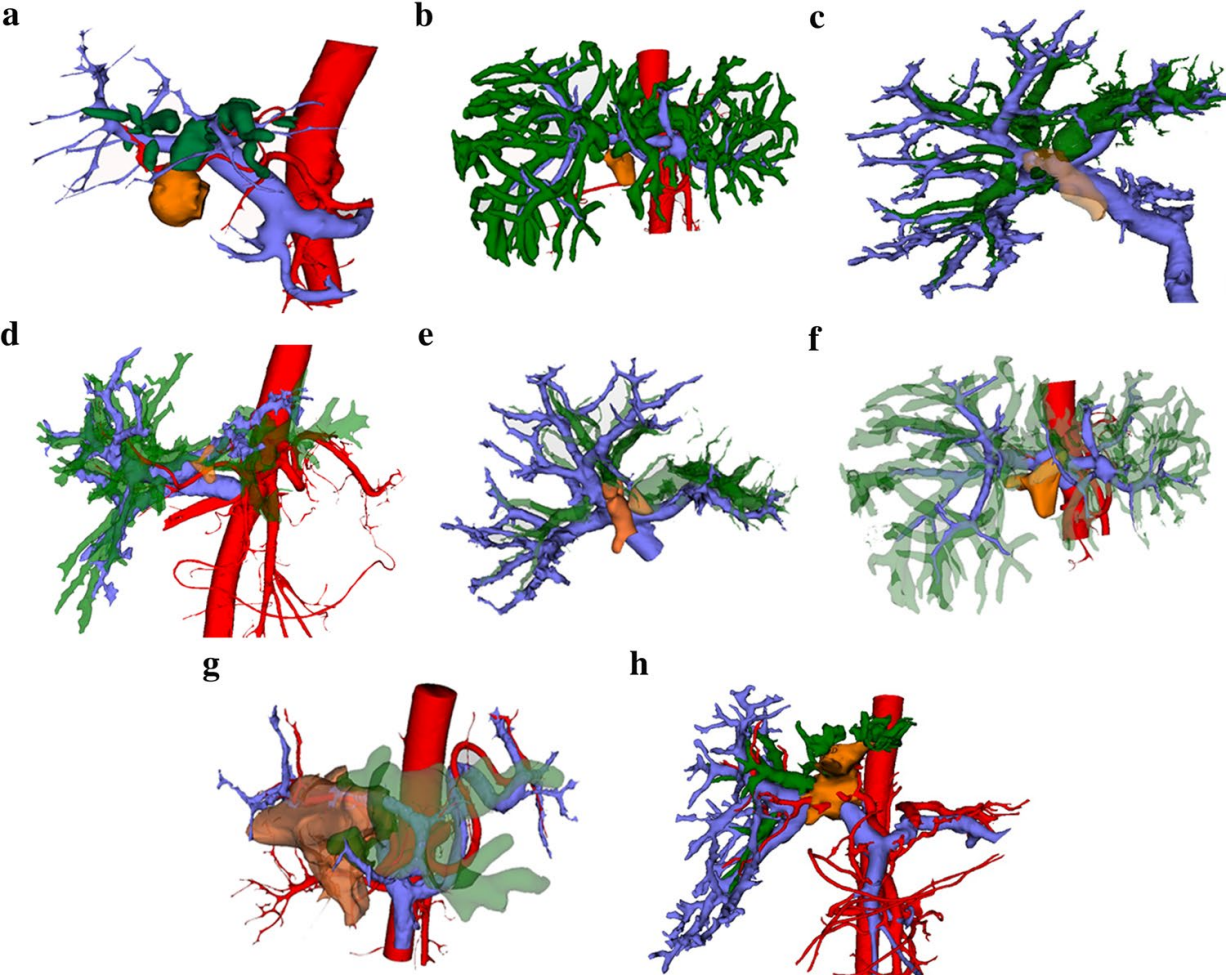
Clinical classification for hilar cholangiocarcinoma based on application of 3D visualization technology. **a** Type I. **b** Type II. **c** Type III a. **d** Type III b. **e**. Type IV a. **f** Type IV b. **g** Type V. **h** Type VI (red for artery; blue for portal vein; green for biliary tract, orange for tumor)

Hilar cholangiocarcinoma is one of the most common biliary malignancies. Surgery is the only treatment for these patients to obtain long-term survival [57]. Generally, Bismuth–Corlette Classification is used to evaluate the extent of tumor invasion in the biliary tract; the MSKCCT grading system is used to evaluate the extent of tumor invasion in the portal vein and the condition of liver atrophy. Combining the advantages of the two methods, we classify hilar cholangiocarcinoma into six types (Fang’s classification) (Table 5; Fig. 4) [25, 58, 59]. Since the characteristics of lymph nodes cannot be defined by 3DVT, this consensus does not involve the content of this field. Through 3DVT analysis of the P and U points (the limit of the dissection points of the portal vein and biliary duct of the normal type, U point: the reverse turn of the horizontal part and sagittal part of the portal vein left branch; P point: the bifurcation of right-anterior branch and right posterior branch of portal vein), the P and U points of the common type of portal vein or the variant portal vein can be observed on 3D [60, 61]. Thus, when type I, II, and III variations occur, the U point is fixed, and the P point moves toward the porta hepatis. Under such circumstances, when hilar cholangiocarcinoma requires extended right hemihepatectomy, we should isolate the trunk of portal vein, right-anterior branch, and left branch, respectively. We resect the right-anterior branch of portal vein when we have completed protecting the trunk and left branch of portal vein. On the other hand, when patients undergo extended left hemihepatectomy, we should isolate the left branch and right-anterior branch of the portal vein, and then resect the left branch of portal vein when we have completed protecting the right-anterior branch of portal vein.

**Table 5:** Three-dimensional visualization clinical classification for hilar cholangiocarcinoma

Type-I: The tumor invades the common biliary duct, and does not invade the confluence part of the right hepatic duct and left hepatic duct, hepatic artery, and portal vein; there is no liver segment or sector atrophy
Type-II: The tumor invades the confluence of right hepatic duct and left hepatic duct, with/without invasion of hepatic artery and/or portal vein, without liver segment or sector atrophy
Type-III a: The tumor invades the confluence of right hepatic duct and left hepatic duct, mainly the right hepatic duct, with invasion of right hepatic artery or right branch of portal vein, with/without right-sided liver sector and/or liver segment atrophy
Type-III b: The tumor invades the confluence of right hepatic duct and left hepatic duct, mainly the left hepatic duct, with invasion of left hepatic artery or left branch of portal vein, with/without left-sided liver sector and/or liver segment atrophy
Type-IV a: The tumor invades the confluence of right hepatic duct and left hepatic duct, the right-sided second-grade biliary duct is involved, with right hepatic artery or right branch of portal vein invasion; tumor has not spread beyond the P point, with right-sided liver segment or liver sector atrophy
Type-IV b: The tumor invades the confluence of right hepatic duct and left hepatic duct, the left-side second-grade biliary duct is involved, with left hepatic artery or left branch of portal vein invasion; the tumor has not spread beyond the U point, with left-sided liver segment or liver sector atrophy
Type-V: The extent of tumor invasion spreads beyond bilateral resection limitation points; right and left hepatic arteries, and left branch and right branch of portal vein are involved, with/without total liver atrophy
Type-VI: The extent of tumor invasion has not spread beyond the P and U points, with involvement of the hepatic artery or portal vein itself or bilateral vessel involvement

Recommendation 16: For patients with hilar cholangiocarcinoma, preoperative 3D clinical classification and evaluation should be performed to guide the selection of appropriate surgical methods (A, Strong recommendation).

## Diagnosis and treatment of hepatolithiasis guided by 3D visualization technology

**Fig. 5:**
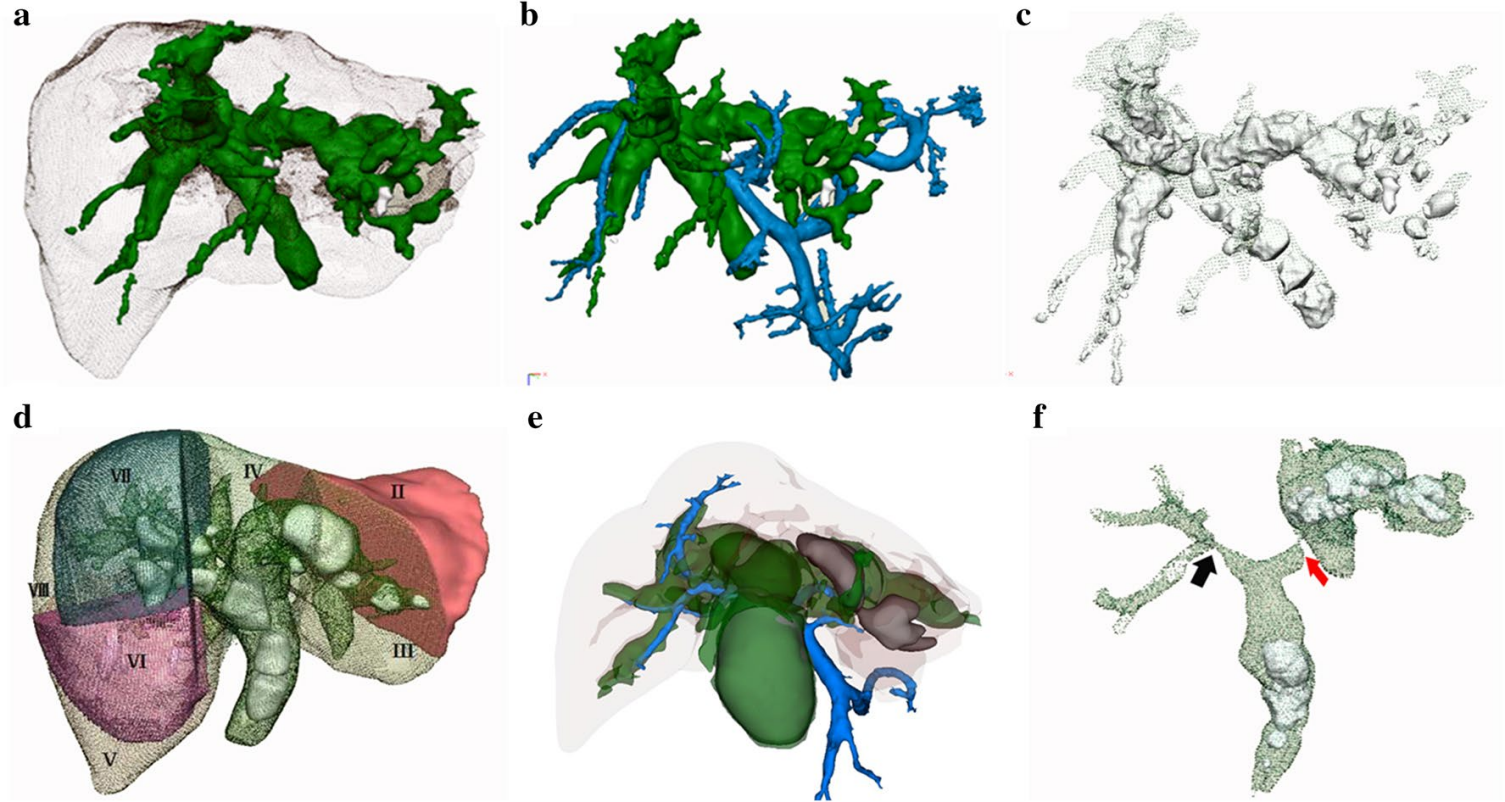
The application of 3D visualization in hepatolithiasis. **a** Transparent liver showed the morphology of the biliary system; **b** the relationship between the biliary system and portal vein was displayed; **c** transparent bile duct showed the distribution, size, and quantity of stones; **d** the distribution of hepatic segments, biliary system, and calculi was shown; **e** the relationship of intrahepatic hepatolithiasis, large common bile duct stones, biliary system, and portal vein was revealed; **f** the distribution of intrahepatic calculi was displayed. Red arrow: obvious dilatation of left hepatic duct, black arrow: relative stenosis of the right hepatic duct

Hepatectomy guided by 3D visualization is a safe and effective method for treatment of hepatolithiasis [61, 62]. In the 3D reconstructed model, the following aspects can be clearly displayed, including the stereoscopic morphology and mutual relationship of “intrahepatic biliary tree” and “vascular tree,” the size and location of stones in the hepatic biliary duct, the degree and extent of bile duct stenosis, the distribution of blood vessels, atrophy or hypertrophy of liver, and the presence of atrophy or variation [33, 63]. The application of 3DVT in hepatolithiasis is demonstrated in Fig. 5. Therefore, in clinical diagnosis, we recommend 3D visualized diagnosis and classification of hepatolithiasis by referring to the stone location (L), biliary stricture (S), biliary dilatation (D), and cirrhosis (C) in the clinical digitized diagnosis of hepatolithiasis (Fang’s classification). For example, hepatolithiasis (LII, LVI, LVII, SII, SVI, SVII, DII, DVI, DVII, and C) showed stones in segments II, VI, and VII, distal biliary dilatation in segments II, VI, VII, and cirrhosis, respectively. However, patients may have hepatolithiasis with or without biliary stricture or biliary dilatation. This digital diagnostic classification can be rapidly generated after the establishment of 3D reconstructed model, which is conducive to the formulation of more reasonable surgical planning [17, 18].

Recommendation 17: 3D visualization should be considered to digital diagnosis and classify hepatolithiasis based on the patient’s clinical status to enhance the stereoscopic comprehension of calculi distribution and pathological bile duct, thereby formulating a more reasonable and radical surgical planning (A, Strong recommendation).

## Guidance of digital minimally invasive surgery in the treatment of hepatolithiasis

Digital minimally invasive surgery refers to the application of digital medical technology such as 3D reconstruction, simulation surgery, and intraoperative navigation in surgery [64]. As long as there is a biliary stent or a drainage tube, 3D visualization technology can be used to guide targeted lithotripsy through a sinus choledochoscope (soft or hard endoscope) to remove residual or recurrent hepatolithiasis; the 3D visualization liver model can be used for overall observation and targeted puncture to avoid tearing of bile duct, injury of hepatic vein, and portal vein [65, 66]. For the elderly or patients with suppurative cholangitis and with physical conditions not allowing the patients to tolerate complex surgical treatment, percutaneous transhepatic choledocholithiasis lithotripsy and stone extraction should be performed under the guidance of 3D visualization technology.

Recommendation 18: Percutaneous transhepatic cholangioscopic lithotripsy (soft or hard endoscope) guided by 3D visualization technology should be considered to formulate more reasonable surgical planning (A, Strong recommendation).

## Diagnosis and management of portal hypertension with 3D visualization

Portal hypertension refers to a clinical syndrome caused by persistent elevation of portal venous pressure. It can be the consequence of liver cirrhosis [67]. HVPG measurement is an internationally recommended gold standard to evaluate the severity of portal hypertension with good repeatability and accuracy. Clinically significant portal hypertension is defined as an increase in HVPG ≥ 10 mmHg (1 mmHg = 0.133 kpa) [68]. It is still difficult to have a dynamic monitoring of clinical outcomes, because HVPG measurement is invasive and costly [69]. At present, a novel non-invasive method for diagnosing portal pressure based on a 3D model combined with computational fluid dynamics (CFD) analysis has been preliminarily applied in clinical practice [10, 70]. In a prospective multicenter study, virtual HVPG based on CT angiography was developed by 3D reconstruction of portal vein models and CFD analysis, and its performance in the non-invasive diagnosis of portal hypertension in cirrhosis was verified. The results demonstrated that virtual HVPG and invasive HVPG showed a significant consistency [71]. The 3D reconstruction of hepatic vascular system based on contrast-enhanced CT can provide a basis for puncture path planning in establishing a transjugular intrahepatic portal vein shunt (TIPS) [72]. A retrospective study reported the feasibility and efficacy of real-time 3D CT image guidance in the TIPS procedure, which could shorten operation time and reduced radiation exposure time. However, the effectiveness of this method needs to be further verified [73].

Recommendation 19: For patients requiring non-invasive or dynamic monitoring of HVPG, it is suggested to perform virtual HVPG to reduce the invasiveness of the procedures if the hospital has an appropriate post-processing workstation (B, Weak recommendation).

Recommendation 20: For patients requiring TIPS, individualized 3D reconstruction is recommended to guide selection of the puncture route (B, Weak recommendation)

## Living donor liver transplantation guided by 3D visualization

Liver transplantation provides an opportunity for definitive treatment for end-stage liver diseases. Living donor liver transplantation (LDLT) has developed rapidly worldwide due to the lack of cadaveric donor livers. The advantages of LDLT include optimized transplant timing, better organ quality, and lower recipient mortality [74, 75]. For LDLT, it is particularly important to clearly understand the volume matching of the graft, as well as the variations in the donor and recipient liver vascular systems. Therefore, accurate preoperative assessment of the donor and recipient liver anatomy is the key factor to the success of the operation and to guarantee the safety of the donor and recipient. In LDLT, 3D reconstruction can accurately and effectively evaluate the liver vascular systems, liver sizes, and volumes of both donor and recipient; simulation surgery allows better resection level selection, vascular processing, and reconstruction of donor liver [8, 76–78]; virtual hepatectomy can improve the success rate of transplantation by optimizing the choice of donor, the decision of venous reconstruction, as well as balancing the situation between the recipient and the donor [79]. The evaluation of bile duct system based on MRI also has important application value in living donor liver transplantation [80]. Venous congestion after LDLT can lead to graft and residual liver failure. Three-dimensional CT reconstruction has been applied to identify the anatomical structure of the hepatic vein with a high risk of graft hyperemia by describing the "regional attribution" of the middle hepatic vein; the liver tissues in the drainage area of the middle hepatic vein can be protected by including the middle hepatic vein in the transplantation graft or reconstructing the vascular drainage [8, 81]. In LDLT for infants or neonates with a limited abdominal space, it is often necessary to reduce the volume of the graft in a complex manner to maintain the necessary vascular structures. Surgeons can simulate donor surgery through 3D images and 3D printed physical models; moreover, the 3D printed model provides a realistic sense of the size, vascular anatomy, and thickness of the reduced graft [52, 78, 82].

Recommendation 21: It is recommended that 3D reconstruction should be performed before LDLT to assess whether there are any variations in the hepatic vascular system in the donor and recipient, and to calculate the size and volume of the graft (A, Strong recommendation).

Recommendation 22: It is suggested that the selection of donor liver resection plane and simulation of the recipient vascular system reconstruction should be carried out through 3D visual simulation surgery to improve the success rate of transplantation (A, Strong recommendation).

## Conclusion

The development of digital intelligent diagnostic and treatment technology has promoted liver surgery from the era of digital anatomy to a new era of digital intelligent diagnostic and treatment [1]. In this consensus, we express our broad and bold vision on the application of 3D visualization technology in liver diseases. At present, digitalized 3D reconstruction and visualized preoperative planning have played an irreplaceable role in liver surgery. We envision that in the reasonably near future, 3D visualization technology will become a routine for the diagnosis and treatment of liver diseases. Standardized 3D visualization processing will eventually make more personalized medical services possible. For this vision to be updated within the routine clinical setting, MDT led by clinicians should be incentivized to participate in this process. Furthermore, standardization is crucial to the success of this endeavor, mainly in the acquisition of high-quality data and 3D reconstruction. Homogenization and standardization are the basis of peer review and clinical practice. At the same time, the design and validation of standardized clinical trial protocols are equally important. These key steps form the foundation for the success of liver 3D visualization technology, which can lead to the routinization of this technology.
